# Determination of Reactive Dyes in Coloring Foodstuff, Fruit Juice Concentrates, and Meat Products by Reductive Azo-Bond Cleavage and LC-ESI-MS/MS Analysis

**DOI:** 10.1021/acs.jafc.4c10320

**Published:** 2025-01-29

**Authors:** Binh Nguyen Thanh, Edwin Januschewski, Wasuki Mahendran, Gerold Jerz, Volker Heinz, Andreas Juadjur, Peter Winterhalter

**Affiliations:** † Institute of Food Chemistry, Technische Universität Braunschweig, Schleinitzstraße 20, 38106 Braunschweig, Germany; ‡ German Institute for Food Technology (DIL), Prof.-von-Klitzing-Straße 7, 49610 Quakenbrück, Germany

**Keywords:** reactive red dye, textile azo dye, food fraud, artificial dyes, reductive azo cleavage, SPE cleanup, HPLC-PDA-ESI-MS/MS

## Abstract

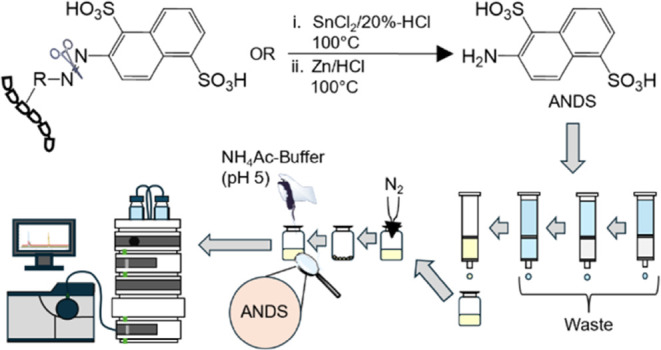

Visually appealing foods are often associated by consumers with subjective quality features, such as freshness, palatability, and shelf life. In the past, there have been repeated violations in which regulations on the use of pigments in food were ignored and/or unauthorized or toxic dyes (e.g., Sudan red in paprika powder) were added. Most recently, adulterations by using reactive dyes from the textile sector have been reported. These included, among others, colored spice preparations for use in meat products (e.g., for sausages and meat products), which were advertised to contain natural plant-derived pigments mainly consisting of betalains and/or anthocyanins. In contrast to natural dyes, reactive dyes are very stable toward extreme pH values, heat, and light. Due to their chemical properties, reactive dyes cannot be detected by classical dye analysis as they may be covalently bound to the food matrix. Methods for the analysis of matrix-bound reactive dyes are therefore required. In this study, a reductive cleavage method of bound textile dyes in different food matrices (spice mixtures, fruit juices, and scalded sausages) and the detection of characteristic cleavage products after enrichment by solid phase extraction (SPE) and liquid chromatography-electrospray ionization-tandem mass spectrometric (LC-ESI-MS/MS) analysis is reported. In addition, 13 suspicious samples provided by project partners were analyzed using the newly developed LC-ESI-MS method.

## Introduction

Natural or artificial food dyes are added to food preparations to compensate for losses during food processing and storage or to increase their attractiveness. Food color is one of the most crucial sensory attributes and is associated with freshness and taste, thus affecting the attractiveness and acceptance of food products.^1^ The use of natural and artificial food colorants is governed by the European Union (EU) Food Additives Directive. According to EU Regulation No. 1333/2008, only colorants that are explicitly permitted in foods can be placed on the market. Currently, 41 natural and synthetic food colors are approved according to Annex II of the EU regulation No 1333/2008. Until the middle of the 19th century, all dyes were obtained from natural sources, such as archil (natural red 28) from lichen, madder (natural red 9) from *Rubia tinctorum*, or Kermes (natural red 3) from *Coccus ilicis*.^2^ The first synthetic dye mauveine was synthesized in 1856 and prepared by William Henry Perkin from coal-tar-based chemicals. A couple of years later, Otto Witt synthesized the azo dye London Yellow.^3,4^ Synthetic dyes are known for their chemical stability, high efficiency, high color strength, low production costs, and broad color spectrum, which is why they are becoming increasingly popular in the food industry.^5^ However, possible adverse effects of synthetic azo dyes, e.g., hyperactivity in children or allergenic, mutagenic, and carcinogenic effects, have led to the banning of numerous synthetic food colorants,^6,7^ such as Sudan I–IV or Rhodamine B. In 1962, the first regulation, “Council Directive 62/2645/EEC” concerning colorants, was issued for the European Economic Community, which was replaced by “European Parliament and Council Directive 94/36/EC”.^8^ Azo dyes, the largest group of synthetic dyes can be readily cleaved by the intestinal flora or human skin bacteria to form aromatic amines, some of which are responsible for carcinogenic or mutagenic activities.^9,10^ Due to these known side effects of various synthetic colorants, a general trend has been observed worldwide that consumers prefer natural pigments in foodstuffs.^11,12^ This trend poses major challenges for food manufacturers due to the often reduced stability, color brilliance, and range of some of the natural dyes and food colorants. Since then, some unscrupulous suppliers have profited from illegal practices by using synthetic colorants that are much more stable than natural pigments. The use of these illegal additives is a form of food fraud and poses potential health risks to consumers. A well-documented example of the unauthorized use of colorings in foodstuffs is the use of Sudan dyes.^13,14^ In 2016, the “Rapid Alert System for Food and Feed” (RASFF) published for the first time a notification concerning the unauthorized use of the textile dye “Reactive Red 195” in fruit pigment extracts. In the same year, a German research group published that anthocyanin- and betalain-based food colorings had been adulterated with the textile dye Reactive Red 195.^15^ Sulfonated reactive dyes are normally in technical use for dyeing of cotton, wool, or polyamide fiber materials. These dyes were discovered by Rattee and Stephan in the year 1954 and were available on the market two years later.^16^ Reactive dyes react chemically with functional groups, such as hydroxyls and amines, and form a covalent bond via reactive anchors. These reactive anchors can be divided into 2 categories: (i) intermediately formed vinyl (exomethylene) groups reacting with polar groups of the matrices through addition to double bonds or (ii) halogen substituents (Cl or F) reacting with textile fibers by nucleophilic substitution. In the textile industry, these dyes are valued for their brilliance, the variety of their color shades, the wash fastness, and their extraordinary variety of applications.^17−19^ These special properties are largely due to their unique reactive groups. If used illegally in food, the reactive dyes can also form covalent bonds with free hydroxyl and/or amino functions of numerous monomeric, oligomeric, and polymeric food ingredients, such as proteins, starches, maltodextrins, or pectins. While approved food dyes can be easily isolated and detected by extraction from the food matrix, this is not possible with these covalently bound textile dyes.^20,21^ The determination of reactive dyes is currently only possible in unbound form.^15,22^ In the bound form, reactive dyes cannot be detected by known methods because they cannot be extracted due to covalent binding.

**Figure 1:**
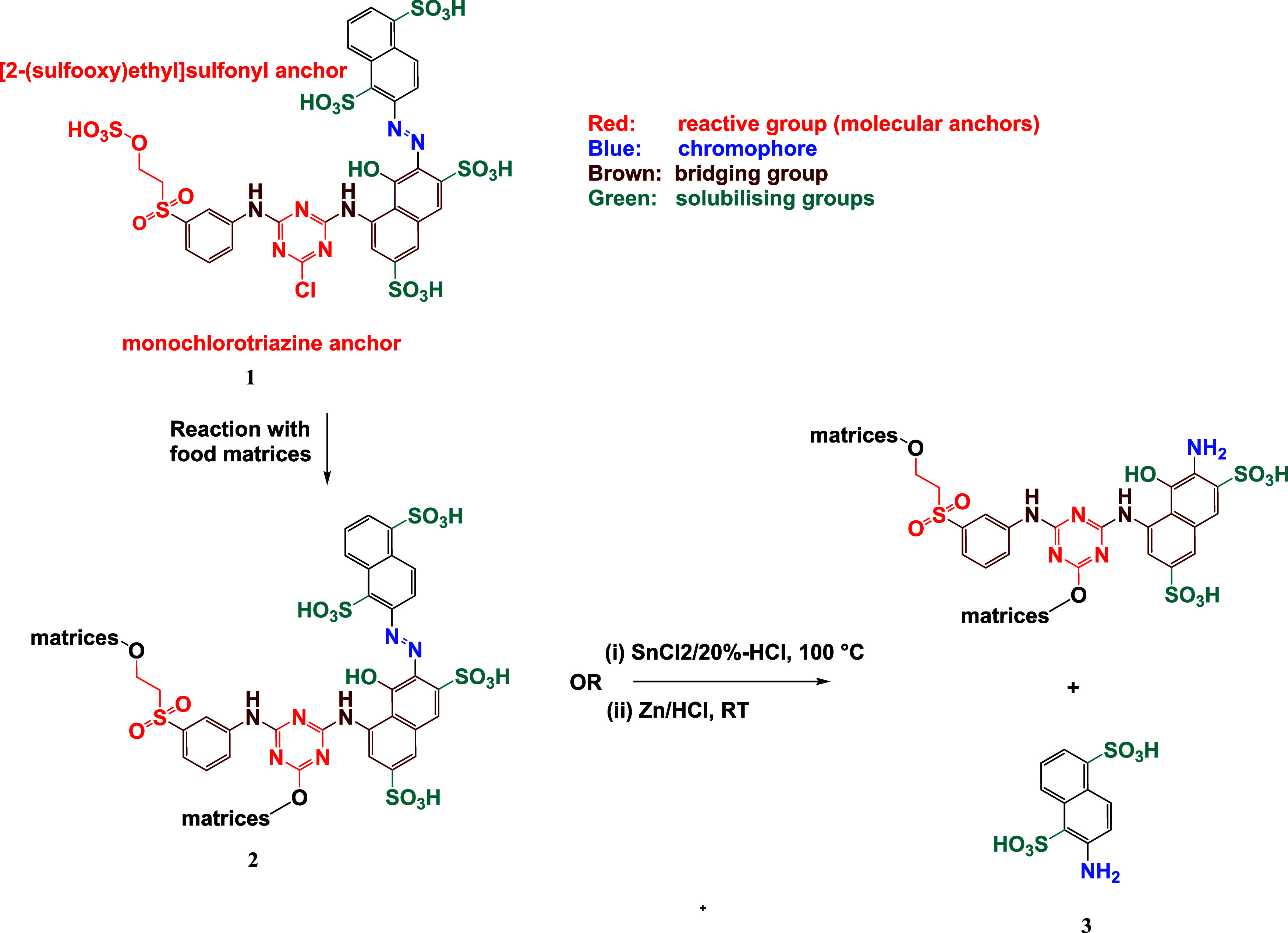
(1) Reaction of Reactive Red 195 with food matrices: Reductive cleavage of matrix linked Reactive Red 195 (2) and its cleavage product 2-amino-1,5-naphthalene-disulfonic acid (3).

One of the possibilities for the detection of bound reactive dyes in food requires the reductive cleavage of the azo-bond moiety (Figure 1). This method is currently mainly used for the detection of azo dyes in consumer goods.^23,24^ Possible reagents for the reductive cleavage are SnCl_2_/HCl or Zn/HCl, which have previously been used to assess the toxicity potentials of azo dyes.^25^ After the reduction, characteristic cleavage products are obtained, which can be used for the detection of an illegal application of textile dyes in food products. In the case of the hydrogenolytic cleavage of the azo bridge in Reactive Red 195 (2), the product 2-amino-1,5-naphthalene-disulfonic acid (3) is released, which can be detected by liquid chromatography-electrospray ionization-tandem mass spectrometric (LC-ESI-MS/MS) analysis after enrichment by solid phase extraction (SPE).

**Figure 2:**
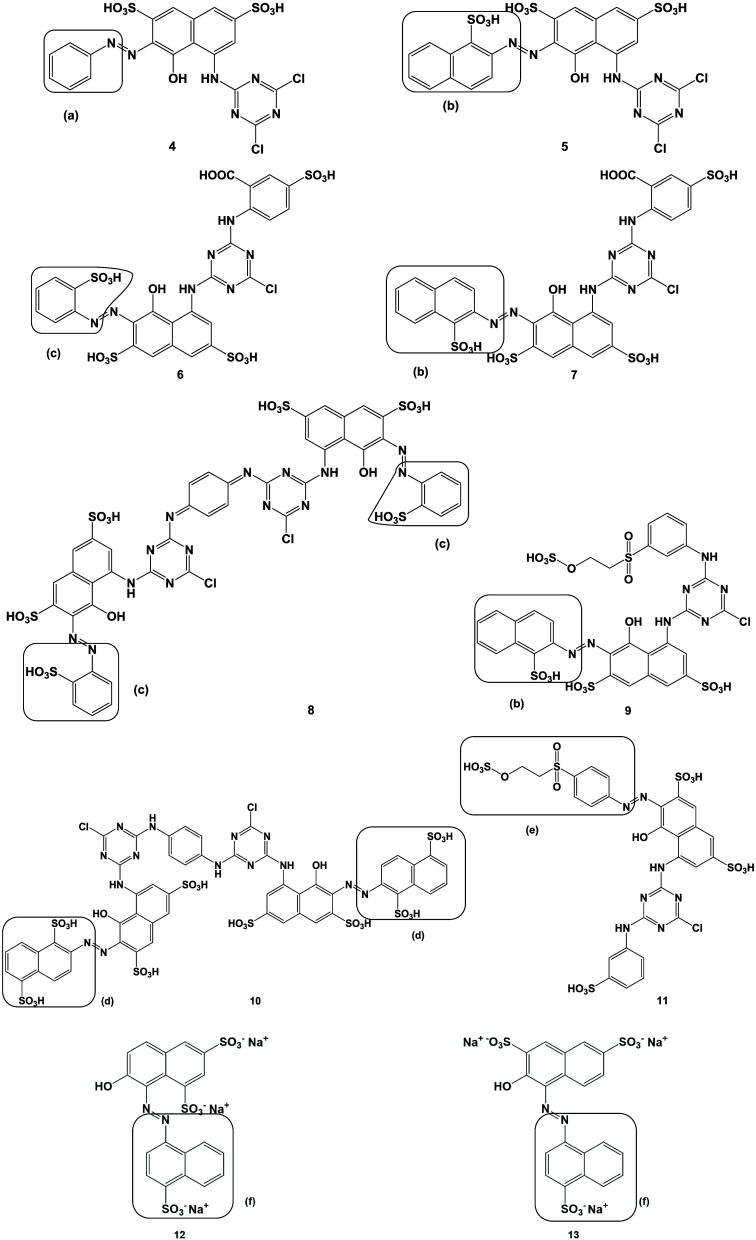
Structural formulas of reactive dyes and artificial food dyes investigated within this study: (4) Reactive Red 2^(I,II)^, (5) Reactive Red 11^(III)^, (6) Reactive Red 29^(I)^, (7) Reactive Red 31^(I,II)^, (8) Reactive Red 120^(I,III)^, (9) Reactive Red 250^(I)^, (10) Reactive Red 141^(I)^, (11) Reactive Red 198^(II,III)^, (12) Amaranth E123^(II)^, and (13) Ponceau 4R E124^(II)^. Cleavage products: (a) aniline (AN), (b) 2-amino-1-naphthalene-sulfonic acid (ANS), (c) 2-amino-benzene-sulfonic acid (ABS), (d) 2-amino-1,5-naphthalene-disulfonic acid (ANDS), (e) 2-((4-amino-phenyl) sulfonyl)-ethyl-hydrogen sulfate (AEHDS), and (f) sodium-4-aminonaphthalene-1-sulfonic acid. Sources: (I) World Dye Variety, (II) Color Index, and (III) Ashford’s Dictionary of Industrial Chemicals.

Figure 2 shows the structures of the reactive dyes and artificial food dyes examined in this study, highlighting the respective indicative cleavage groups used for analytical detection by LC-ESI-MS/MS. In the following section, the newly developed method for the detection of textile dyes in food is described and applied to the analysis of reactive dyes. In this study, commercial samples and also laboratory-made food samples were spiked with reactive dyes for qualitative analysis.

## Materials and Methods

### Chemicals and Reagents

Ultraclean water was prepared by Nanopure Diamond Analytical with a pore size of 0.2 μm from Wilhelm Werner (Leverkusen, Germany). Sn-(II)-chloride (anhydrous for synthesis), Casein from bovine milk (technical grade) 2-amino-1-naphthalene-sulfonic acid (>98%), 2-amino-benzene-sulfonic acid (95%), and 2-amino-1,5-naphthalene-disulfonic acid were purchased from Sigma-Aldrich (Taufkirchen, Germany). Aniline (≥99.5%) was purchased from Carl Roth (Karlsruhe, Germany). Zinc powder (for analysis, particle size <45 μm), ammonia (25%, p.a.), acetic acid, and hydrochloric acid were purchased from Merck (Darmstadt, Germany). Acetonitrile (LC-MS grade) was purchased from Honeywell Specialty (Seelze, Germany). Ammonium acetate (LC-MS grade) and potassium disulfite powder were purchased from VWR Chemicals (Fontenay-sous-Bois, France). Protease A was purchased from the Tokyo Chemical Industry–TCI (Tokyo, Japan). Formic acid (LC-MS grade), petrol ether (analytical grade), and methanol (HPLC grade) were purchased from Fisher Chemicals (Pittsburgh, Pennsylvania, USA). Raw white wool for binding experiments of reactive dyes was purchased from Wolle Rödel GmbH & Co., KG (Paderborn, Germany).

### Reference Dyes and Samples

Reactive red dyes were obtained from various manufacturers. Spice mixtures produced on a laboratory scale with color additives were provided by the German Institute for Food Technology (DIL, Quakenbrück, Germany). Juice concentrates were provided by Symrise AG (Holzminden, Germany) and meat products were provided by DIL as well as Meat Cracks Technologies (Steinfeld, Germany).

### HPLC-PDA-ESI-MS/MS

The HPLC system (1100/1200 series, Agilent, Waldbronn, Germany) consisted of a binary pump (G1312A), an autosampler (G1329A), and a PDA detector (G1316A) and was coupled to an ion-trap mass spectrometer (HCT Ultra ETD II, Bruker Daltonics, Bremen, Germany) with an electrospray ionization source (ESI). The samples were separated on a ProntoSIL 120–5 C18 AQ column with precolumn (250 mm × 2.0 mm, Bischoff chromatography, Leonberg, Germany) using water +1.0% formic acid (eluent A) and acetonitrile +1.0% formic acid (eluent B) at a flow rate of 0.25 mL/min. The gradient steps were set from 99% A to 80% A in 20 min, to 50% A in 15 min, to 0% A in 10 min, at 0% A for 10 min, to 99% A in 5 min, and at 99% A over 10 min. The column temperature was set to 20 °C. The ESI source was operated in positive and negative modes using nitrogen as a nebulizer (60 psi) and drying gas 11.0 L/min (temp. 350 °C). The scan range was set between *m*/*z* 50 and 1000 using the rapid detection *Ultra mode* with a mass scanning range of 26.000 *m*/*z* per second. MS-Parameters were as given: voltage at HV capillary 3500 V, HV end plate offset −500 V, trap drive 57.9, octopole Rf amplitude 153.2 Vpp, lens 2 60 V, capillary exit −109.1 V, target mass 302, max. accumulation time 200.000 μs, ICC target 70,000, average 5 spectra and precursor ions 3, and fragmentation amplitude 1 V. Ten microliters of the sample was analyzed by HPLC-PDA-ESI-MS/MS and the results were evaluated with Data Analysis 3.0 (Bruker Daltonics, Bremen, Germany).

### Sample Preparation

#### Reductive Cleavage of Reactive Dye Solutions with SnCl_2_/HCl and Zn/HCl

For the identification of the cleavage products, standard solutions of reactive dyes were treated with SnCl_2_/HCl and Zn/HCl, respectively. 1.5 mL of the standard solution (2.0 g/L) was transferred to a V-shaped vial (Supelco, Sigma-Aldrich, Missouri) for reduction with SnCl_2_/HCl, mixed with 1.0 mL of a SnCl_2_ solution (250 mg/mL) in 20% aqueous hydrochloric acid, and heated in a heating block (Techne Ori-Block DB-1) at 100 °C until complete decolorization. The colorless samples were processed by SPE (Supelco SPE vacuum chamber, Sigma-Aldrich, Missouri) with Strata-X-AW (500 mg/6 mL, Phenomenex Ltd., Aschaffenburg, Germany) and Strata-X-C (500 mg/6 mL, Phenomenex Ltd., Aschaffenburg, Germany). The eluates were membrane-filtered (pore size: 0.45 μm, Macherey & Nagel, Düren, Germany) and analyzed by LC-ESI-MS/MS according to the method described above. For zinc reduction, 250 mg of elementary zinc powder and 200 μL of HCl were added to 2.0 mL of the standard solution in a test tube and then vigorously shaken. The eluates were degassed by ultrasonication, membrane-filtered (pore size: 0.45 μm), and analyzed by LC-ESI-MS/MS.

#### Color-Labeled Casein and Wool for Model Reactions

In the case of casein, 5 g of this milk protein was mixed with 100 mg of the respective reactive dyes in 30 mL of water and stirred for 24 h at ambient temperature. The stained samples were passed over a pleated filter and washed with water until the filtrate remained colorless. The wool threads, previously degreased with petroleum ether, were immersed in a reactive dye solution (1.5 g/L) and left to stand overnight at ambient temperature. The wool threads were then rinsed with water to remove residual unbound reactive dye material and dried at ambient temperature.

#### Reductive Cleavage of Dyed Casein and Wool Threads

For the reduction of the artificially colored casein, 2 g of the sample was mixed with 0.5 g protease A and 15.0 mL of 10 mmol acetic acid-ammonium acetate buffer (pH 8). The solution was incubated at 50 °C for 6 h. For reduction, 1.0 mL of SnCl_2_/20%-HCl solution (250 mg/mL) was added and then heated at 100 °C for 2 h until decolorization occurred. Two grams of the dyed wool threads were mixed with 10 mL of water and 1.0 mL of a 250 mg/mL SnCl_2_/20%-HCl solution and heated at 100 °C until decolorization. The colorless samples were centrifuged at 11.000 rcf for 10 min (Universal 30 F centrifuge, Hettich GmbH & Co., KG, Tuttlingen, Germany), and the residue was washed 3 times with 10 mL of water. The supernatants were combined and processed by SPE (material cf. above). For the reduction with zinc, 2 g of casein and wool threads were mixed with two solutions of 250 mg/500 mg zinc in 200 μL/400 μL HCl, respectively.

#### Reductive Cleavage of Color-Labeled Preparations

Spice mixtures spiked with reactive dyes were prepared at DIL (Quakenbrück, Germany). 0.5 g of color-labeled products was dissolved in 15.0 mL of water and treated in an ultrasonic bath for 5 min. Then, 1.0 mL of SnCl_2_/20%-HCl (250 mg/L) solution was added to the sample and heated at 100 °C until decolorization. The colorless samples were centrifuged at 11.000 rcf for 10 min, and the residue was washed three times with 10 mL of water. The supernatants were combined and processed by SPE.

#### Reductive Cleavage of Color-Labeled Fruit Concentrates

The fruit juice concentrates were diluted until the minimum Brix value (cf. Table 1) according to Annex 6 Regulation on fruit juice, fruit nectar, and caffeinated soft drinks (Fruchtsaft- and Erfrischungsgetrankeverordnung-FrSaftErfrischGetrV). The Brix value was determined with a digital refractometer (Hanna instruments, Nuşfalău, Romania). Various red-approved and nonapproved colorants were added to the fruit juices for analysis.

**Table 1:** Minimum Brix Values [deg] for Reconstituted Fruit Juice and the Added Colorants

fruit concentrate	brix [deg] of fruit juice	colorants
cranberry (*Vaccinium macrocarpon*)	10.0	RR195
bilberry (*Vaccinium myrtillus*)	11.2	RR120
blackberry (*Rubus sect. rubus*)	11.1	carmine
raspberry (*Rubus idaeus*)^a^	7.0	RR11
lingonberry (*Vaccinium vitis-idaea*)	10.3	E124

a Annex 6 FrSaftErfrischGetrVO.

For decolorization of natural anthocyanins in fruit juices, 3 g of the sample was mixed with 40 mL of potassium disulfite solution (0.5 mol/L) and was kept at room temperature for 20 min. For chemical reduction to analyze added reactive dyes, 1.0 mL of SnCl_2_/20%-HCl (250 mg/mL) solution was added and heated at 50 °C until complete decolorization. The treated samples were centrifuged at 11.000 rcf for 20 min, and the residue was washed three times with water. The supernatants were combined and processed by SPE.

#### Reductive Cleavage of Sausages and Meat Products SnCl_2_/HCl

Sausage and meat products were prepared by DIL (Quakenbrück, Germany) as well as Meat Cracks Technologie GmbH (Steinfeld, Germany). Samples were minced and freeze-dried (β 2–8 LD Plus, Martin Christ Gefriertrocknungsanlagen, Osterode am Harz, Germany). The freeze-dried samples were placed in extraction thimbles and extracted with petrol ether using Soxhlet extraction (Soxtherm Gerber, Königswinter, Germany) for approximately 6 h. The sleeves were dried in a drying oven (50 °C) for several hours. Three grams of the dried and defatted sample was mixed with 0.5 g protease A and 20.0 mL of acetic acid-ammonium acetate buffer (10 mmol, pH 8.0). The solution was incubated at 50 °C for 6 h. For reduction, 1.0 mL of SnCl_2_/20%-HCl solution (250 mg/mL) was added and then heated at 100 °C until decolorization. The colorless samples were centrifuged at 11.000 rcf for 20 min, and the residue was washed three times with 10 mL of Nanopure water. The supernatants were combined and processed by SPE, for LC-ESI-MS/MS analysis.

### Solid Phase Extraction (SPE) Using Weak Anion-Exchange Cartridges (Strata-X-AW)

Conditioning of the Strata-X-AW (500 mg/6 mL, Phenomenex Ltd., Aschaffenburg, Germany) was accomplished by passing 4.0 mL of MeOH and 4.0 mL of water through the cartridges. The sorbent was not allowed to dry, and the prepared sample was loaded. The washing step was performed by adding 8.0 mL of water and 8.0 mL of MeOH. The elution step was performed by adding 4.0 mL of 5% methanolic NH_3_ solution. The eluates were dried under a N_2_ stream, and the residues were redissolved in 10.0 mL of acetic acid-ammonium acetate buffer (10 mmol, pH 5.0). Subsequently, the samples were membrane-filtered and analyzed by LC-ESI-MS.

### Solid Phase Extraction (SPE) Using Strong Cation-Exchange Cartridges (Strata-X-C)

Conditioning of the Strata-X-C (500 mg/6 mL, Phenomenex Ltd.) was accomplished by passing 4.0 mL of MeOH and 4.0 mL of 0.1 N HCl through the cartridges. The sorbent was not allowed to dry, and the prepared sample was loaded. The washing step was performed by adding 8.0 mL of 0.1 N HCl and 8.0 mL of 0.1 N HCl in MeOH. The elution step was performed by adding 4.0 mL of 5% methanolic NH_3_ solution. The eluates were dried under a N_2_ stream, and the residues were dissolved in 10.0 mL of acetic acid-ammonium acetate buffer (10 mmol, pH 5.0). Subsequently, the samples were membrane-filtered and analyzed by LC-ESI-MS.

## Results and Discussion

### Reduction of Reactive Dye Solutions and Dyed Casein and Wool Threads with SnCl_2_/HCl and Zn/HCl

The reduction of reactive dye standard solutions, dyed casein, and dyed wool threads was carried out until complete decolorization. The required reduction time had to be adjusted in each specific case. For example, the RR195 sample was colorless within 15 min with SnCl_2_/HCl, whereas RR120 required up to 120 min until complete decolorization. Reduction with Zn/HCl took slightly longer compared to SnCl_2_/HCl cleavage. Decolorization of the dyed wool threads and casein with SnCl_2_/HCl took, on average, 30 min longer compared with the dye solutions. Reduction with SnCl_2_/HCl worked well for all matrix-bound reactive dyes, but zinc reduction did not lead to a color shift in casein samples and wool threads, even after an extended reaction time of 24 h and a doubling of the amount of zinc. This clearly shows that the food matrix has a major influence on the reducing power of the zinc powder and thus makes it more difficult to cleave the azo bridge in the reactive dyes. After reduction with SnCl_2_/HCl, the samples were processed by SPE to purify the cleavage products. Since the pH values of the samples were not within the working range (pH 2–10) of the used C18-LC column, most of the reducing agent SnCl_2_ precipitated as Sn(OH)Cl in alkaline or as well in a slightly acidic medium. Due to its poor solubility in water, it precipitates as a white sediment, which could have hampered the SPE procedure. It turned out that the amount of reducing agent used for the reduction of the dyes did not affect the enrichment process and was removed via the SPE washing steps.
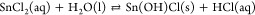
1

**Figure 3:**
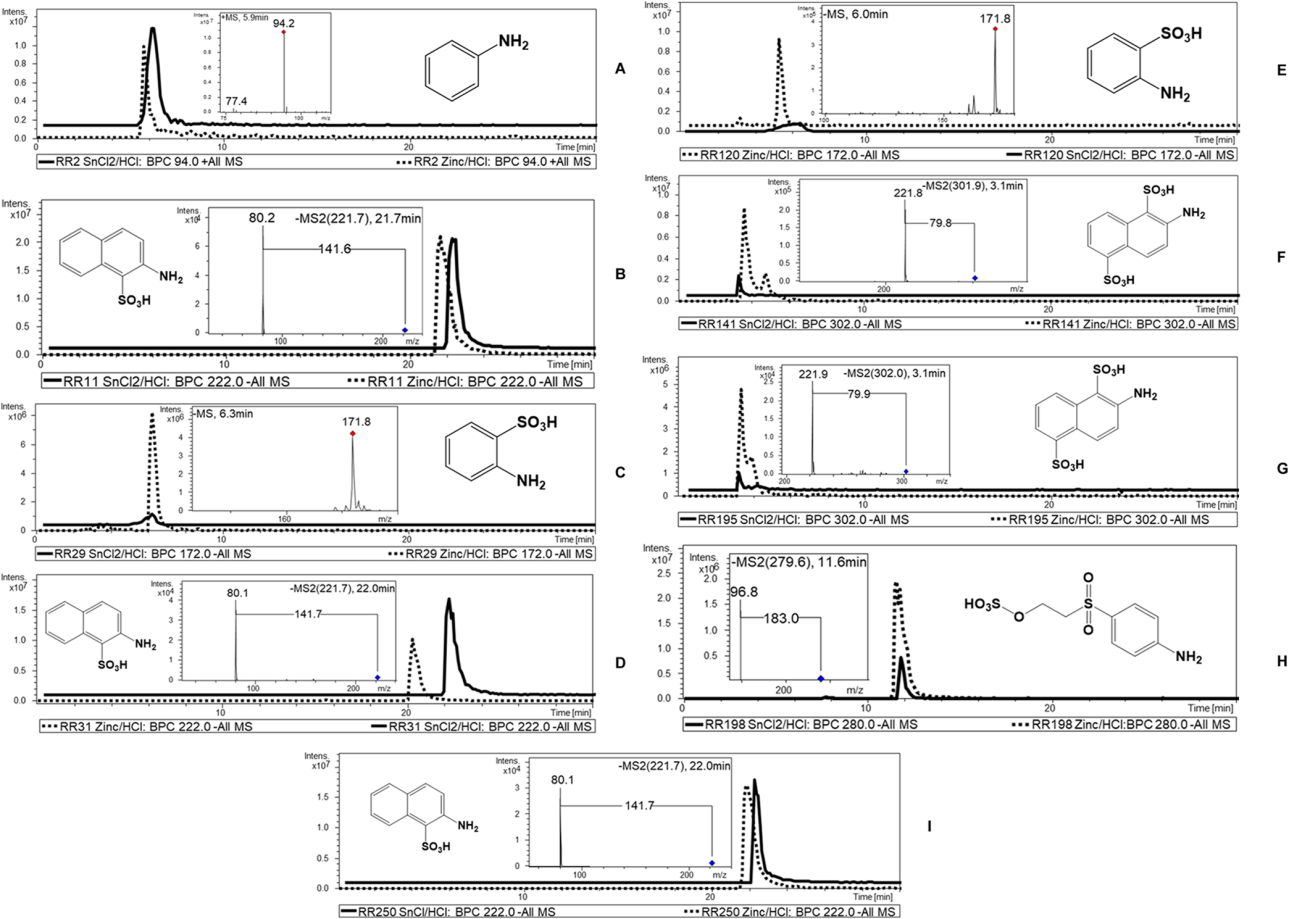
LC-ESI-MS/MS chromatograms with the target ion isolation of cleavage products generated from dye solutions reduced with SnCl_2_/HCl. Pos. mode: (A) RR2. Neg. mode: (B) RR11. (C) RR29. (D) RR31. (E) RR120. (F) RR141. (G) RR195. (H) RR198. (I) RR250.

The enrichment of the target analytes was carried out by using SPE cartridges, i.e., Strata-X-AW and Strata-X-C. Strata-X-AW is a weak anion-exchange functionalized polymeric sorbent for the enrichment of acidic compounds with a p*K*_a_ less than 5 and was used for the purification of sulfonated compounds, such as 2-aminonaphthalene-1,5-disulfonic acid (ANDS). However, the enrichment of aromatic amines, such as aniline, was not successful with this sorbent material. For aromatic amines, the strong cation-exchange functionalized polymeric sorbent material Strata-X-C was more effective for SPE and can be applied for basic compounds with p*K*_a_ values less than 10.5. Figure 3 shows the LC-ESI-MS/MS chromatograms of the reduced and purified dye solutions. The hydrogenolytic reaction with RR2 led to the cleavage product aniline (AN) with [M + H]^+^ at *m*/*z* = 94. In the case of the dyes RR11, RR31, and RR250, the reaction led to the target cleavage products 2-amino-naphthalene-1-sulfonic acid (ANS) with [M – H]^−^ at *m*/*z* 172, and for RR29 and RR120 to 2-amino-benzene-sulfonic acid (ABS) ([M – H]^−^*m*/*z* 222), respectively. The cleavage of the dyes RR141 and RR195 resulted in the formation of the disulfonated product 2-amino-naphthalene-1,5-disulfonic acid (ANDS) (cf. Table 2). The MS/MS spectral data of chemically cleaved metabolites revealed the neutral loss difference of Δ*m*/*z* 80 for a sulfate unit and indicated the sulfonate substitution.

**Table 2:** LC-ESI-MS/MS Data of the Reactive Dye Cleavage Products

	reactive dye	*R_t_* (min)	LC-ESI-MS/MS	cleavage product
A	Reactive Red 2	5.9	[M + H]^+^*m*/*z***94**	AN
B	Reactive Red 11	21.7	[M – H]^−^*m*/*z***222** MS^2^ 80	ANS
C	Reactive Red 29	6.3	[M – H]^−^*m*/*z***172**	ABS
D	Reactive Red 31	22.0	[M – H]^−^*m*/*z***222** MS^2^ 80	ANS
E	Reactive Red 120	6.0	[M – H]^−^*m*/*z***172**	ABS
F	Reactive Red 141	3.1	[M – H]^−^*m*/*z***302** MS^2^ 222	ANDS
G	Reactive Red 195	3.1	[M – H]^−^*m*/*z***302** MS^2^ 222	ANDS
H	Reactive Red 198	11.6	[M – H]^−^*m*/*z***280** MS^2^ 222	AEHDS
I	Reactive Red 250	22.0	[M – H]^−^*m*/*z***222** MS^2^ 80	ANS

The highlighted cleavage products of the investigated reactive dyes (cf. Figure 3) were also detected in the dyed casein samples and wool threads after reduction with SnCl_2_/HCl. Especially for the LC-ESI-MS/MS analysis of bound reactive dyes in casein samples, a previous proteolytic digestion step of the protein matrix with *Aspergillus oryzae* protease was required. This additional processing improved the SPE cleanup as the strong interference by protein precipitation was omitted. Without the proteolytic digestion step, significantly fewer cleavage products were detected, as reductive cleavage reagents might get deactivated or used in side reactions.

The model experiments with casein and wool threads clearly demonstrated that bound reactive dyes can be detected by LC-ESI-MS/MS analysis via their cleavage products, and the method is therefore likely to be suitable for the analysis of real food samples. Nevertheless, the identification of individual dyes based on the cleavage products is limited in certain cases because different reactive dyes produce identical products after azo-bridge cleavage. An example is the formation of ANDS, which can be released from the reactive dyes RR141 or RR195 (Cf. Figure 3F,G).

In contrast to many other dyes already mentioned above, RR198 (cf. Figure 2-11) is classified as a bifunctional reactive dye. An important structural difference is that one of the two reactive anchors is directly attached to the cleavage product, and no release from the covalently bound matrix could be achieved. Reductive cleavage of RR198 in aqueous solution led to the identification of 2-((4-amino-phenyl)-sulfonyl)ethyl-hydrogen sulfate in the hydrolysate using LC-ESI-MS/MS analysis (cf. Figure 3H). However, as expected, this cleavage product could not be detected in the dyed casein and wool thread samples because the target metabolites are still bound to the matrix. Hence, this method is not suitable for the dye RR198 and all other bifunctional reactive dyes with reactive anchors located on two structural elements separated by an azo bond, as the release of a cleavage product is not possible. The method development for the analysis of bifunctional dyes is part of an ongoing project and will be published later.

### Validation of the Method

A blind sample validation was performed to evaluate the analytical method. The aim was to verify the reliability of the developed method for identifying reactive dyes and to detect food samples that are not marketable or have been treated with illegal dyes. For this purpose, coloring spice preparations (4 samples CP-1–4), fruit juices (5 samples FJ-5–9), and cooked sausages (6 samples SA-10–15) were selected as representative food samples for the stress test (cf. Table 3). Some of these real food samples were mixed with approved and nonapproved dyes, respectively, and analyzed in our laboratories using the SnCl_2_/HCl method. In a double-blinded approach, neither the group that prepared the labeled food samples nor the group performing the chemical analysis were informed about the identity of the dyes added to the samples. The dyes were bottled under coded labels, the prepared samples were provided with new codes, and the coding algorithm was sealed in an envelope and dissolved after the analyses.

**Table 3:** Prepared Samples for Blind Validation Analysis

	sample	dye		sample	dye
CP-1	coloring preparation 1	E123	SA-10	sausage 157	RR222
CP-2	coloring preparation 2	RR11	SA-11	sausage 268	RR11
CP-3	coloring preparation 3	RR222	SA-12	sausage 356	E123
CP-4	coloring preparation 4	red beet	SA-13	sausage 429	E123
FJ-5	blackberry juice	E120	SA-14	sausage 623	RR222
FJ-6	cranberry juice	RR195	SA-15	sausage 701	RR11
FJ-7	bilberry juice	RR120			
FJ-8	raspberry juice	RR11			
FJ-9	lingonberry juice	E124			

### Analysis of Coloring Spice Preparations

**Figure 4:**
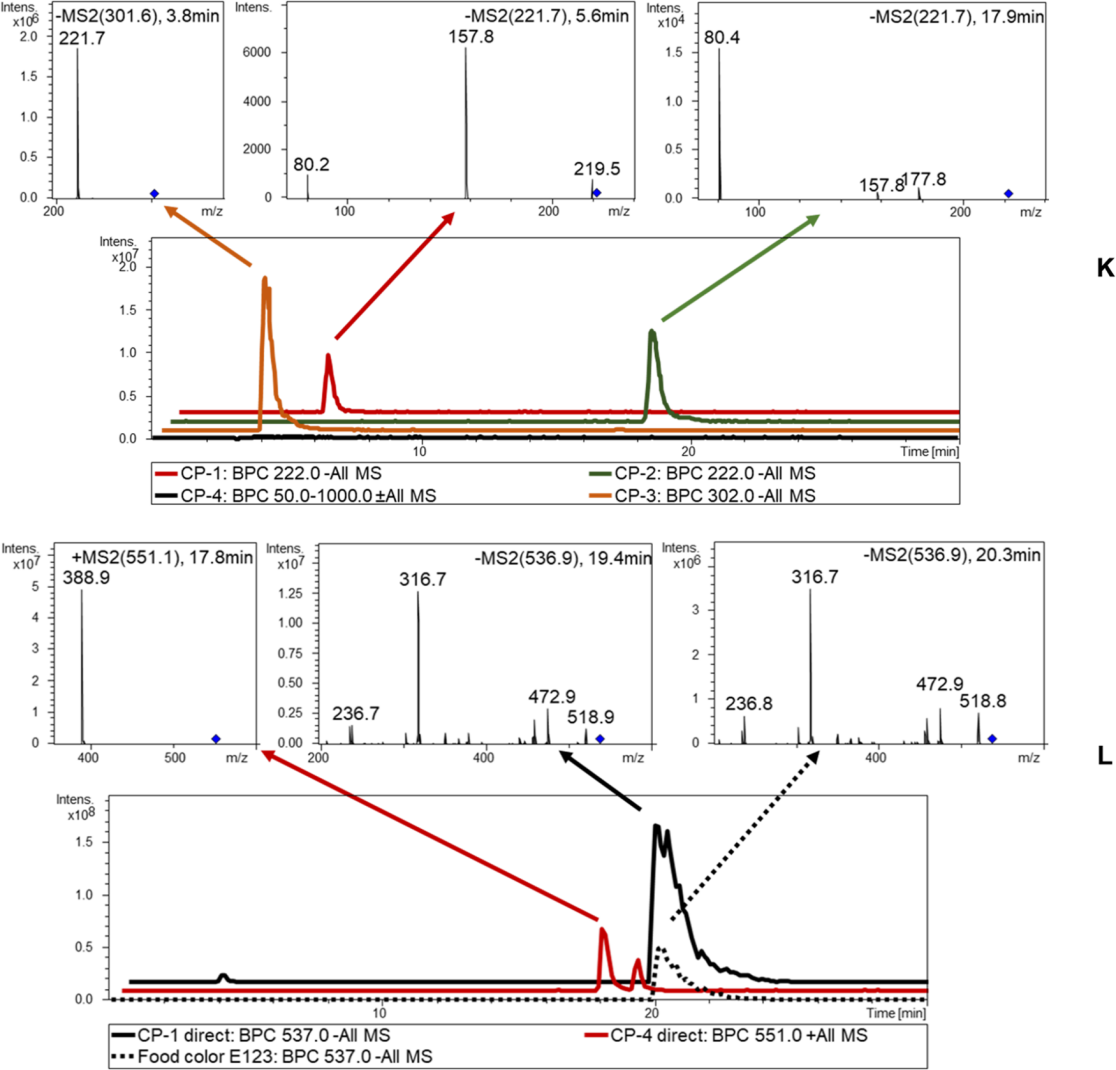
LC-ESI-MS chromatogram of the coloring preparations. (K) Coloring preparations after reductive cleavage. (L) Coloring preparations 1 (E123) and 4 (betanin/iso-betanin) without reductive SnCl_2_/HCl cleavage.

In the unknown coloring preparation 1 (CP-1, Table 3) after reductive cleavage, LC-ESI-MS/MS analysis revealed a peak with *m*/*z* 222, indicating ANS, a cleavage marker compound known for reactive dyes. However, compared to the authentic ANS sample, the compound showed a different retention time on C-18-LC-ESI-MS. Further direct examination of the colored aqueous extract by LC-ESI-MS without reductive SnCl_2_/HCl cleavage reaction revealed the molecular ion signal [M – H]^−^ at *m*/*z* 537, indicating the presence of the azo dye E123 (cf. Figure 4). The result was confirmed by coinjection of E123 with and without cleavage reaction, resulting in identical MS/MS patterns and retention time values. In this case, the use of the reductive cleavage initially led to a misleading result because the approved artificial azo dyes (E123) released the constitutional isomer of 2-amino-naphthalene-1-sulfonic acid (ANS) with identical molecular weight (*m*/*z* 222). Only the different C-18-LC-ESI-MS retention time compared to the respective mass-value of the chemically generated isobar with differing sulfonyl-substitution patterns from the reactive dyes RR11, RR31, and RR250 enables the detection of E123 via its cleavage product.

In samples CP-2 and CP-3 (cf. Table 3), the sulfonated compounds 2-amino-naphthalene-1-sulfonic acid (ANS) and 2-amino-naphthalene-1,5-disulfonic acid (ANDS) were detected after reductive cleavage. However, it was not possible to clearly characterize the individual dyes because other reactive dyes may also produce the same cleavage products. Nevertheless, it can be said with certainty that an artificial azo dye was added, which clearly refutes the claim that natural colorants were added. For example, the reactive dyes RR11, RR31, or RR250 are candidates for liberating ANS, and the dyes RR141, RR195, and RR222 are candidates for liberating ANDS. It should also be noted that only a small selection of reactive dyes was used in this study, and it cannot be ruled out that other dyes may produce the same cleavage products.

After reductive cleavage, no known cleavage products were detected in the spice mixture CP-4. Furthermore, no sulfonated compounds with a characteristic mass loss of Δ*m*/*z* 80 were observed in the LC-ESI-MS chromatogram, indicating that no artificial azo dyes were used. By analyzing the spice mixture CP-4 without using the SnCl_2_/HCl reductive cleavage, characteristic signals for betanin and iso-betanin at *m*/*z* 551 [M + H]^+^ (cf. Figure 4) were detected with MS/MS for betanidin at *m*/*z* 389 by loss of the glucose unit, indicating the addition of coloring materials from beetroot (*Beta vulgaris*). Hence, direct LC-ESI-MS/MS analysis of the colored samples is a necessary complementation to investigate the presence of natural pigments (such as betalains and anthocyanins) or artificial nonreactive azo dyes, such as E123 next to reactive dyes.

### Analysis of Fruit Juices

**Figure 5:**
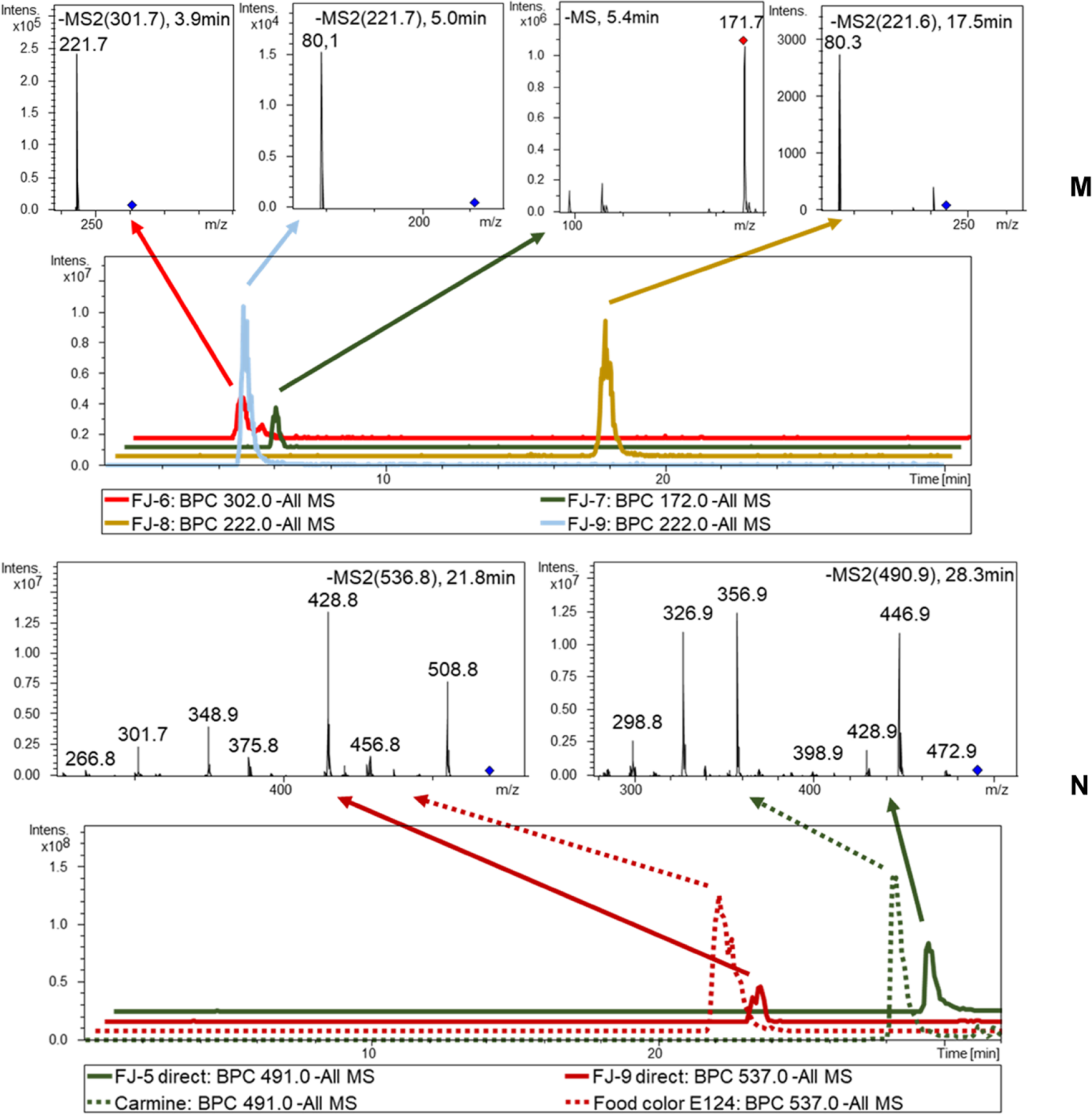
LC-ESI-MS/MS chromatograms of the fruit juice samples. (M) Fruit juices (sample FJ-6–8) were obtained after reductive SnCl_2_/HCL cleavage. (N) Fruit juices (samples FJ-5 and FJ-9) contained carmine and E123 without reductive SnCl_2_/HCl cleavage.

The addition of reactive dyes was reliably detected in three out of the five fruit juice samples, namely, cranberry (FJ-6), bilberry (FJ-7), and raspberry juice (FJ-8) (cf. Table 3), via the typical cleavage products, which are generated by the SnCl_2_/HCl reaction. ANDS was detected as a cleavage product in cranberry, ANS in raspberry, and ABS in bilberry. In Lingonberry (FJ-9), the constitutional isomer of ANS was observed in the LC-ESI-MS/MS chromatogram (cf. Figure 5). In the case of the fruit juice samples, the reduction temperature had to be adjusted from 100 to 50 °C in order to prevent the nonenzymatic browning reaction (Maillard reaction). The resulting brown melanoidin pigments interfered with the visual assessment of the SnCl_2_/HCl reduction end point. High concentrations of red and purple anthocyanins had to be decolorized by the addition of potassium metabisulfite (K_2_S_2_O_5_) prior to the reduction step.^26^ In the lingonberry (FJ-9) and blackberry juice (FJ-5) samples, the colorants E124 and carmine were identified by the wool thread adsorption method.

### Analysis of Scalded Sausages

**Figure 6:**
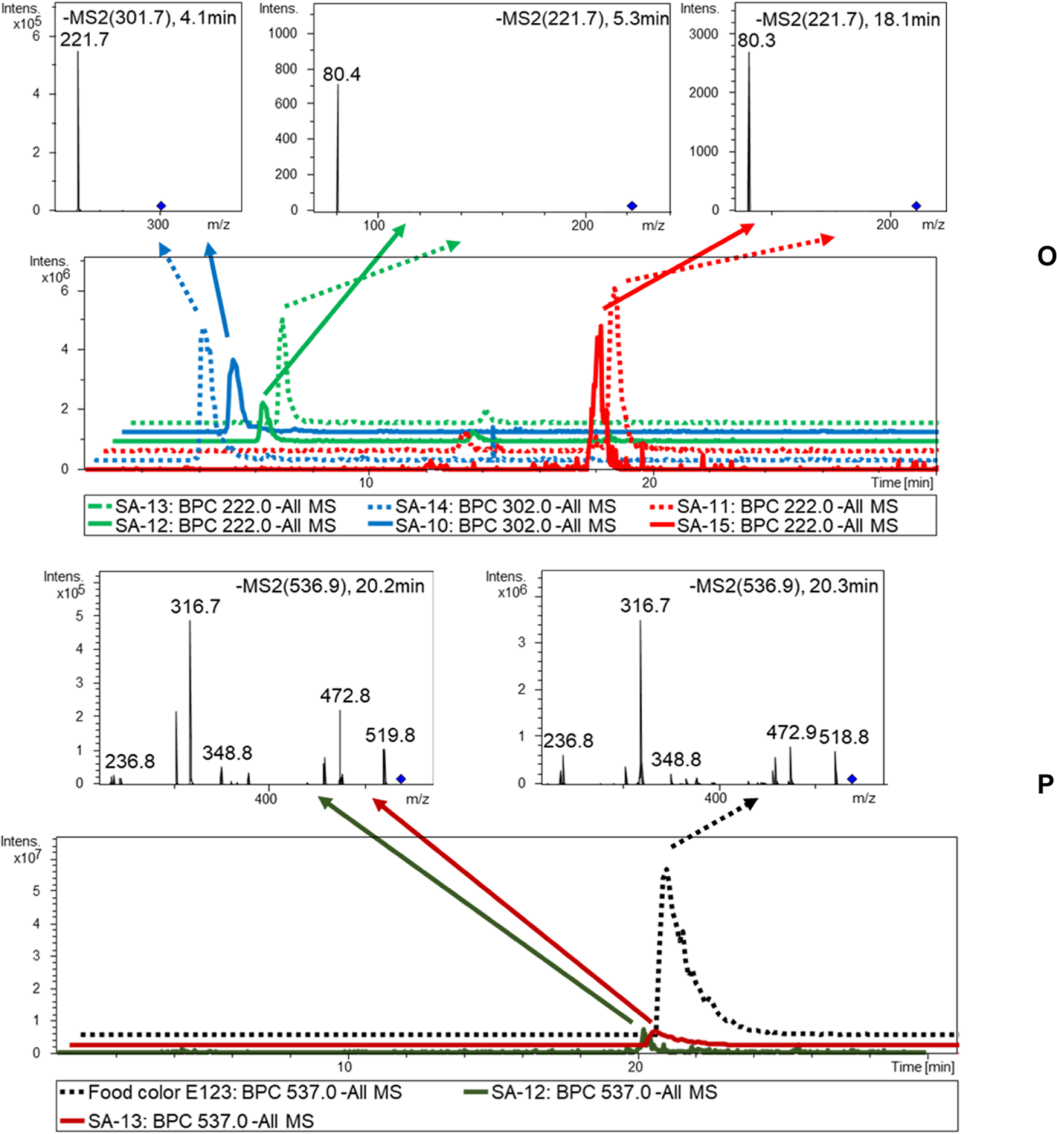
LC-ESI-MS/MS chromatogram of a sausage sample. (O) The sausage was served after SnCl_2_/HCl reductive cleavage. (P) Sausage and E123 without reductive cleavage.

The addition of reactive dyes has been reliably demonstrated in sausages. In samples SA-11 and SA-15, the SnCl_2_/HCl cleavage reaction generated the product 2-amino-naphthalene-1-sulfonic acid [M – H]^−^ at *m*/*z* 222 as seen in the LC-ESI-MS/MS analysis (cf. Figure 6). As a result of azo-bond cleavage, the samples SA-10 and SA-14 displayed the product ion at [M – H]^−^*m*/*z* 222 for 2-amino-naphthalene-1,5-disulfonic acid. In the sausage samples SA-12 and SA-13, the lower concentration of the nonreactive azo dye E123 was detected after previous processing with the wool thread adsorption method.

Prior to the SnCl_2_/HCl reductive cleavage step of the reactive dyes in the sausage matrix, the entire meat material had to be subjected to a proteolytic digestion step with Protease A. This step eliminated the protein matrix, which negatively influenced the SnCl_2_/HCl reduction activity and also hindered the SPE adsorption process of the indicative target molecules, such as, e.g., ANDS and ABS for LC-ESI-MS/MS detection.

### Application of the Analytical Method to Samples Suspected of Containing Reactive Dye Additives

Sixteen samples (SP-A–SP-M) suspected of illegal dye additives were purchased outside of the European Union and supplied by various companies with confidential identities. According to these manufacturers, the samples were labeled as containing only natural colorants that were said to have high color strength as well as thermal and acid stability, which is generally atypical for legal natural pigment classes for use in food. To analyze the suspect samples, the LC-ESI-MS/MS method was used to determine reactive dyes and other natural dyes, such as betalains and anthocyanins. Of the 16 suspected samples, reactive dyes could be reliably detected in 13 dye preparations (cf. Table 4). After reductive cleavage, the liberated products ANDS (SP-A, SP-F, SP-G, SP-H, SP-K, SP-L, and SP-M), ABS (SP-I and SP-J), and AEHDS (SP-B, SP-C, SP-D, and SP-E) were identified in the samples. The results of the rapid photometric method RaSDAY^22^ confirmed the illegal use of reactive dyes in these suspicious samples.

**Table 4:** Results of Suspect Samples by LC-ESI-MS/MS and RaSDAY

	suspicious preparation	LC-ESI-MS/MS	compound	RaSDAY^22^	reactive dye
SP-A	coloring preparation A	[M – H]^−^*m*/*z***302** MS^2^ 222	ANDS	artificial dyes	+
SP-B	coloring preparation B	[M – H]^−^*m*/*z***280** MS^2^ 97	AEHDS	artificial dyes	+
SP-C	coloring preparation C	[M – H]^−^*m*/*z***280** MS^2^ 97	AEHDS	artificial dyes	+
SP-D	coloring preparation D	[M – H]^−^*m*/*z***280** MS^2^ 97	AEHDS	artificial dyes	+
SP-E	coloring preparation E	[M – H]^−^*m*/*z***280** MS^2^ 97	AEHDS	artificial dyes	+
SP-F	coloring preparation F	[M – H]^−^*m*/*z***302** MS^2^ 222	ANDS	artificial dyes	+
SP-G	coloring preparation G	[M – H]^−^*m*/*z***302** MS^2^ 222	ANDS	artificial dyes	+
SP-H	coloring preparation H	[M – H]^−^*m*/*z***302** MS^2^ 222	ANDS	artificial dyes	+
SP-I	coloring preparation I	[M – H]^−^*m*/*z***172**	ABS	artificial dyes	+
SP-J	coloring preparation J	[M – H]^−^*m*/*z***172**	ABS	artificial dyes	+
SP-K	coloring preparation K	[M – H]^−^*m*/*z***302** MS^2^ 222	ANDS	artificial dyes	+
SP-L	coloring preparation L	[M – H]^−^*m*/*z* MS 222	ANDS	artificial dyes	+
SP-M	coloring preparation M	[M – H]^−^*m*/*z***302** MS^2^ 222	ANDS	artificial dyes	+

The aim of the research project was to develop a suitable analytical method for the sensitive detection of covalently and matrix-bound reactive dyes in food matrices. The focus of the investigation was on spice extracts with coloring properties and fruit juice concentrates, as well as the detection of such illegal dyes in meat products (i.e., sausage). The newly developed method consists of a reductive cleavage reaction of samples/food sources by SnCl_2_/HCl, which results in specific degradation products released from reactive dyes, which are then identified by LC-ESI-MS/MS. This mass spectrometric detection of the cleavage products, in combination with the published rapid photometric RaSDAY method, is a further tool for validating the existence and specifying the identity of covalently bound reactive dyes in all kinds of food samples. During the project, we received a number of suspicious samples, which were analyzed using the developed LC-ESI-MS/MS method. Reactive dyes were detected in 13 out of 16 suspicious samples, highlighting the ongoing problem of the illegal use of reactive textile dyes in foods and the analytical need for reliable detection methods of these illegal azo dyes in complex food matrices. Quantification of the cleavage products was not the main focus of these studies, as the amount of cleavage products is irrelevant for positive confirmation. However, our investigations have also shown that reductive cleavage is not suitable for quantification under the mentioned conditions. It can be concluded that an error in the measuring device is not the cause of the issue, as the “intraday” precision determined for the degradation products ANDS, ANS, and ABS analyzed was within an acceptable range for chromatographic methods (*V*_k_ < 1%). However, the “interday” precision demonstrated that the degradation products (ANDS *V*_k_ = 12%; ANS, ABS *V*_k_ = 4%) do not exhibit adequate stability over several days (6 days). One potential explanation for the suboptimal stability of the decomposition products over several days is the presence of the reducing agent SnCl_2_/HCl. The addition of the reducing agent immediately led to a significant reduction in the recovery rate of the cleavage product ABS from just 10%. The instability of the degradation products in relation to the reducing agent was also observed for ANDS and ANS. With regard to these two degradation products, no reduction in the recovery rate was observed immediately after the addition of the reducing agent. However, a continuous decrease in the recovery rate was observed during the reduction. Consequently, it can be concluded that failure to adhere to the exact reduction time can lead to large deviations in the measurement results. In the future, the parameters of the reductive cleavage should be adjusted with respect to quantification.

## Supporting Information Available

List of reference dyes (Table S1); LOD/LOQ of cleavage products (Table S2); overview of the analysis of reactive dyes in different food products (Figure S1); and LC-ESI-MS/MS chromatogram of suspicious samples (Figure S2) (PDF)

## Abbreviations and Nomenclature

RR: reactive red
ANS: 2-amino-1-naphthalene-sulfonic acid
ABS: 2-amino-benzene-sulfonic acid
ANDS: 2-amino-1,5-naphthalene-disulfonic acid
AN: aniline
AEHDS: 2-((4-amino-phenyl)-sulfonyl)-ethyl-hydrogen sulfate
