# Supplementary material for: Determination of Reactive Dyes in Coloring Foodstuff, Fruit Juice Concentrates, and Meat Products by Reductive Azo-Bond Cleavage and LC-ESI-MS/MS Analysis
Source: J Agric Food Chem. 2025 Jan 29;73(6):3703–13. doi: 10.1021/acs.jafc.4c10320 (PMC11826996; doi:10.1021/acs.jafc.4c10320)
Supplement: Supplementary file 1 — jf4c10320_si_001.pdf [file jf4c10320_si_001.pdf]

Supporting Information

Determination of Reactive Dyes in Coloring Foodstuff, Fruit Juice Concentrates and Meat Products by Reductive Azo-bond Cleavage and LC-ESI-MS/MS Analysis

Binh Nguyen Thanh [x], Edwin Januschewski [x, y], Wasuki Mahendran [x], Gerold Jerz [x], Volker Heinz [y], Andreas Juadjur [y], Peter Winterhalter [x*]

[x] Institute of Food Chemistry, Technische Universität Braunschweig, Schleinitzstraße 20, 38106 Braunschweig, Germany

[y] German Institute for Food Technology (DIL), Prof.-von-Klitzing-Straße 7, 49610 Quakenbrück, Germany

* corresponding author: p.winterhalter@tu-bs.de

Table S1. List of reference dyes

Reactive Dye	*Providing company*
RR2	Sigma-Aldrich Chemie GmbH, Taufkirchen, Germany
RR11-1	FastColours LLP, Huddersfield, UK
RR11-2	Molekula GmbH, München, Germany
RR11-3	Undisclosed provider 1
RR29	Sigma-Aldrich Chemie GmbH, Taufkirchen, Germany
RR31	Undiclosded provider 1
RR120-1	FastColours LLP, Huddersfield, UK
RR120-2	Undisclosed provider 2
RR141	Undisclosed provider 2
RR195-1	Undisclosed provider 2
RR195-2	Undisclosed provider 3
RR198-1	FastColours LLP, Huddersfield, UK
RR198-2	Undisclosed provider 2
RR198-3	Molekula GmbH, München, Germany
RR250	Undisclosed provider 1

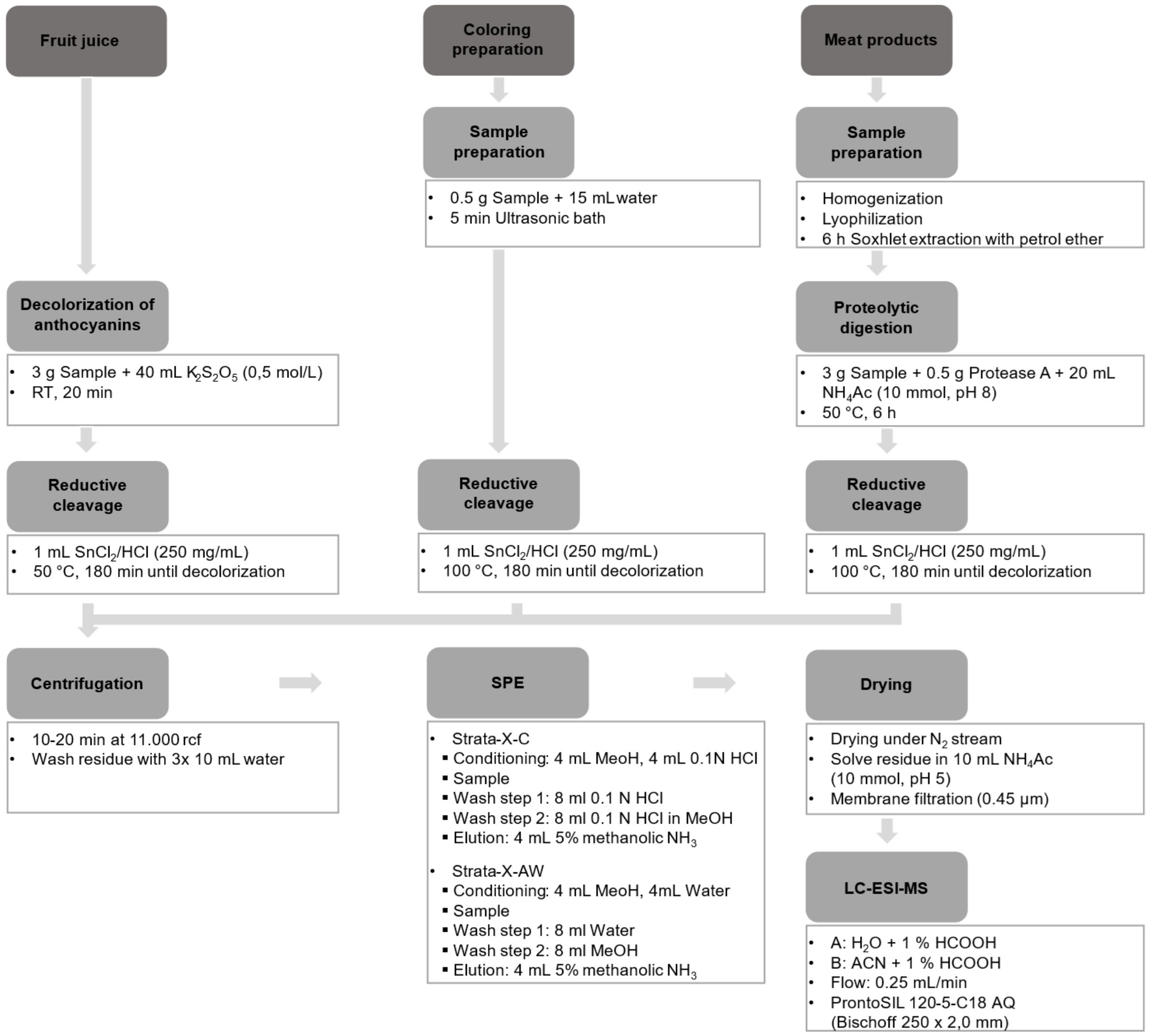

Figure S1. Overview on the analysis of reactive dyes in different food products.

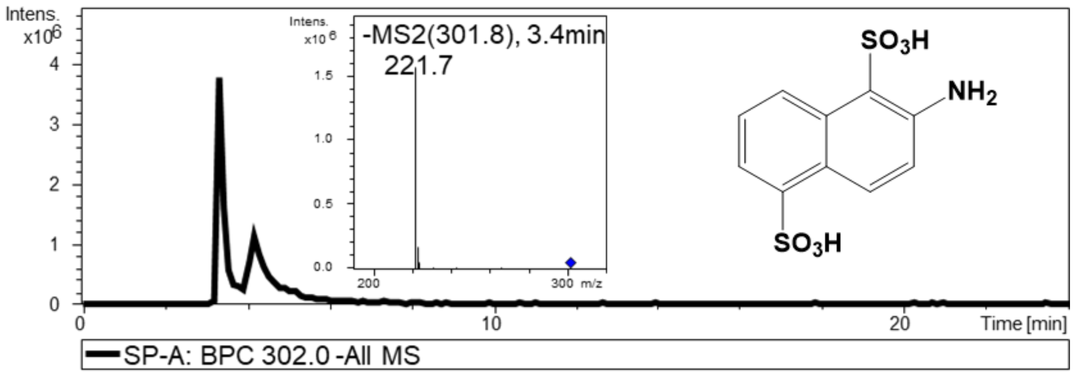

A1

17

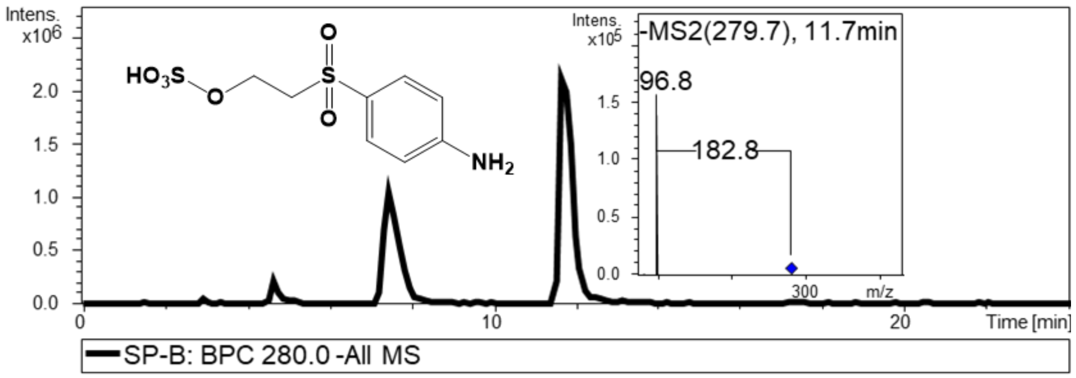

B1

18

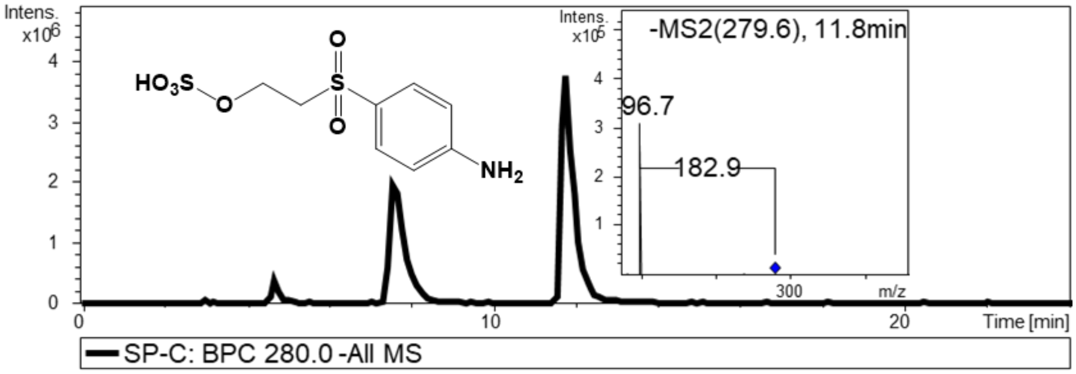

C1

19

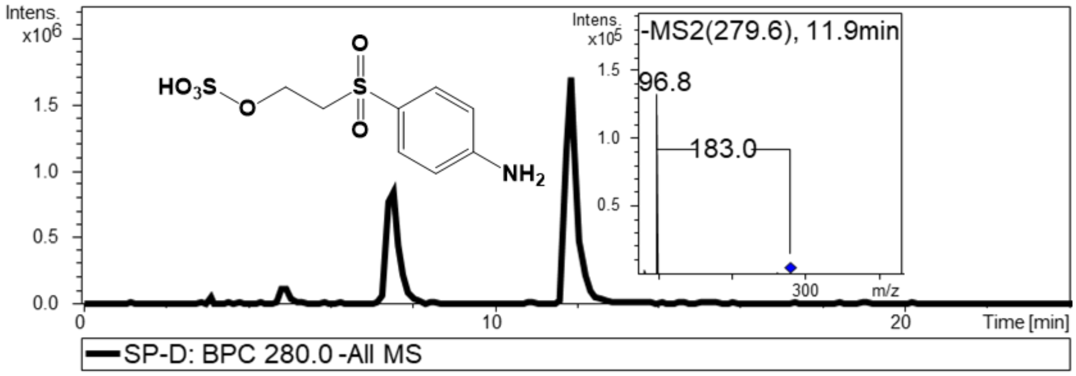

D1

20

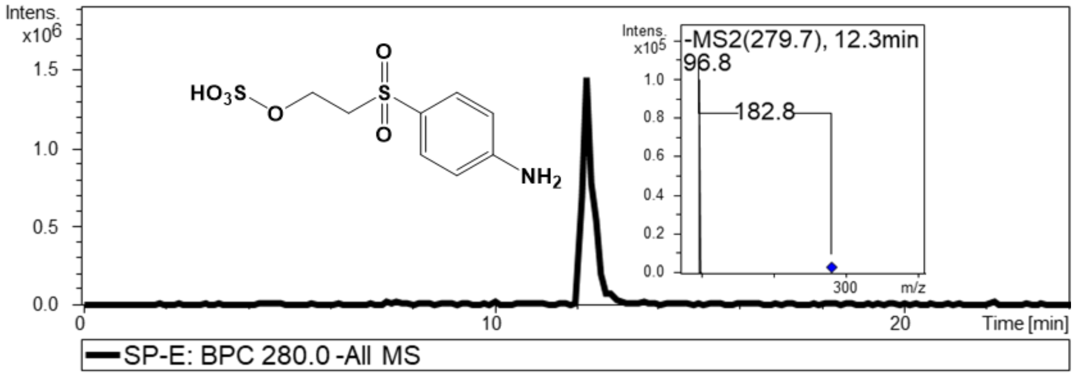

21

E1

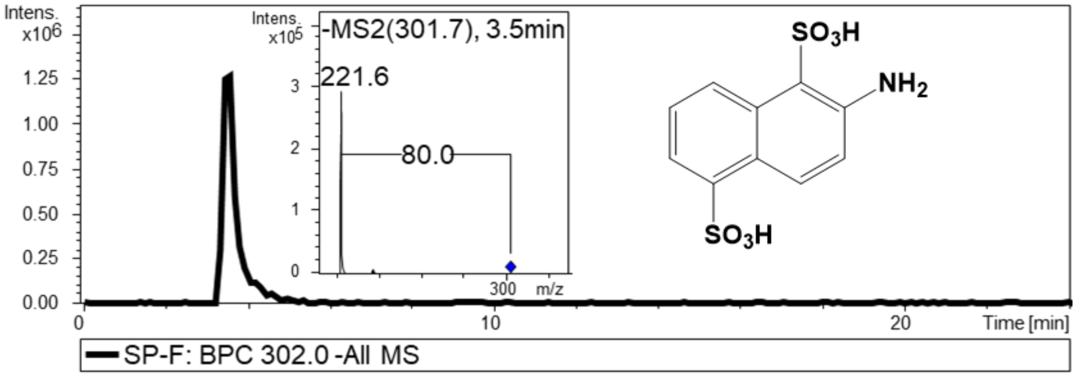

22

F1

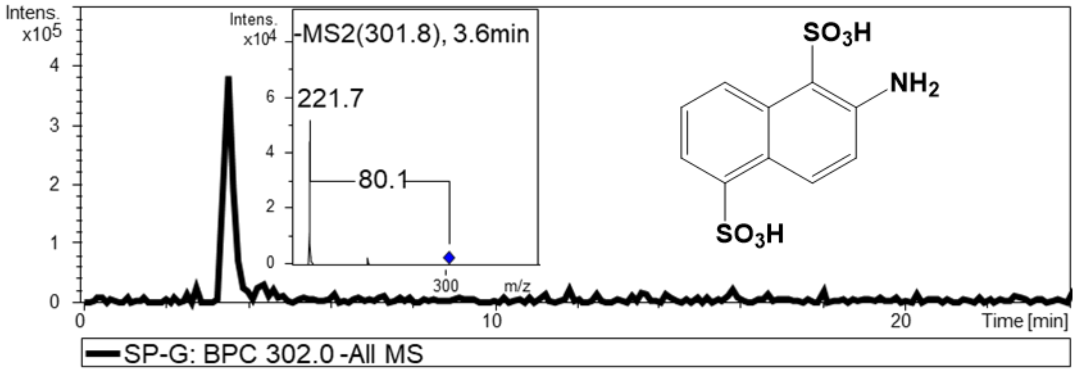

23

G1

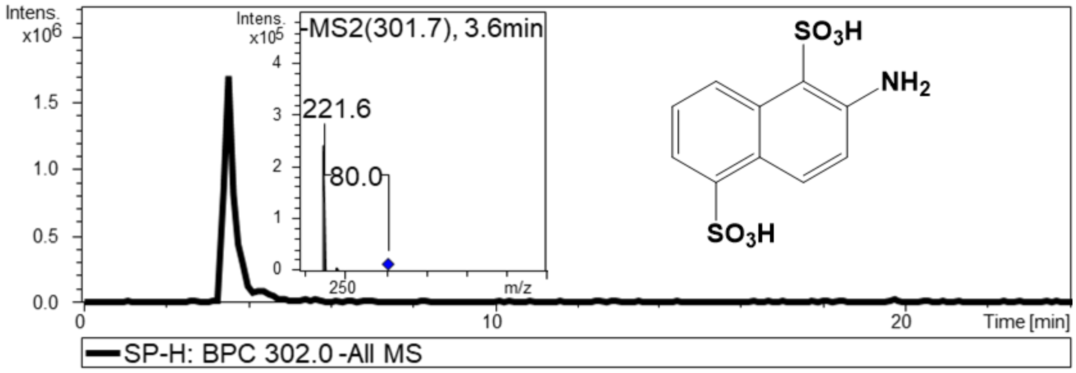

24

H1

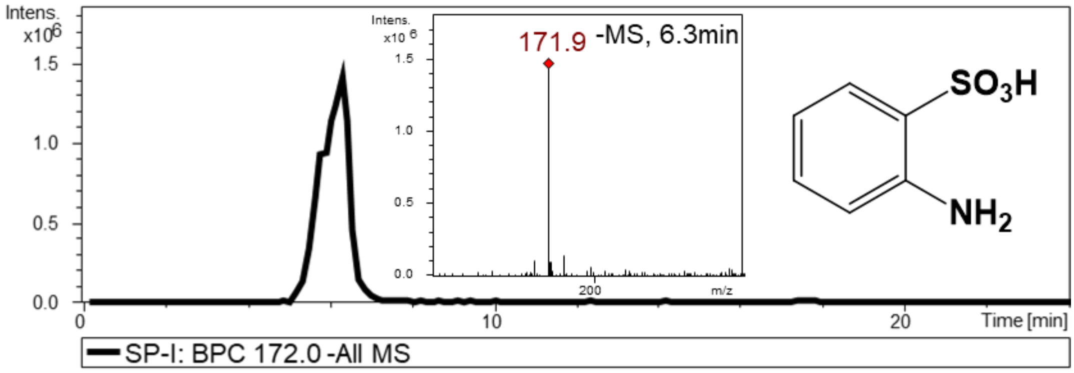

25

I1

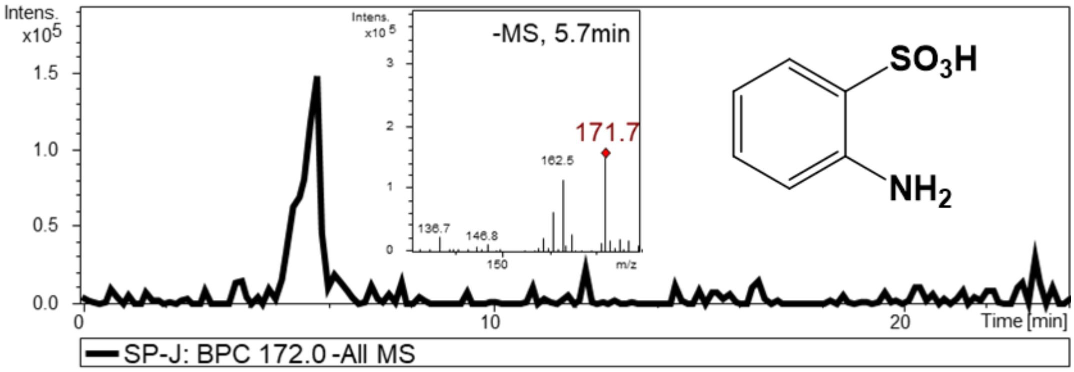

26

J1

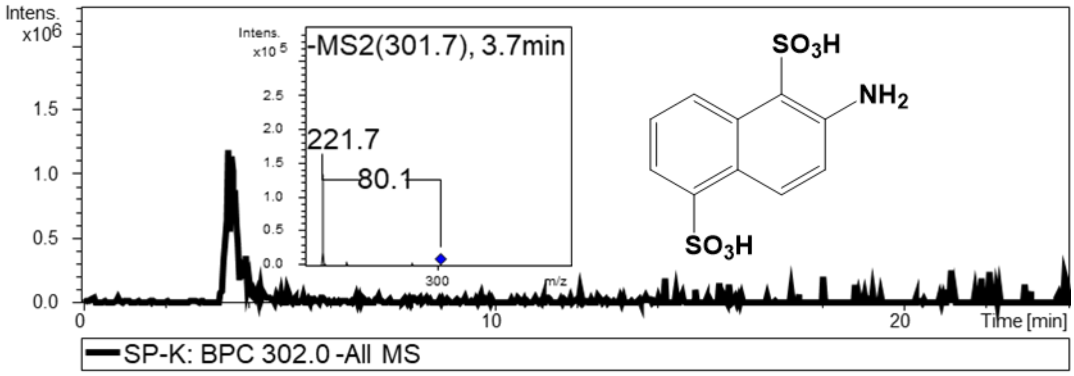

27

K1

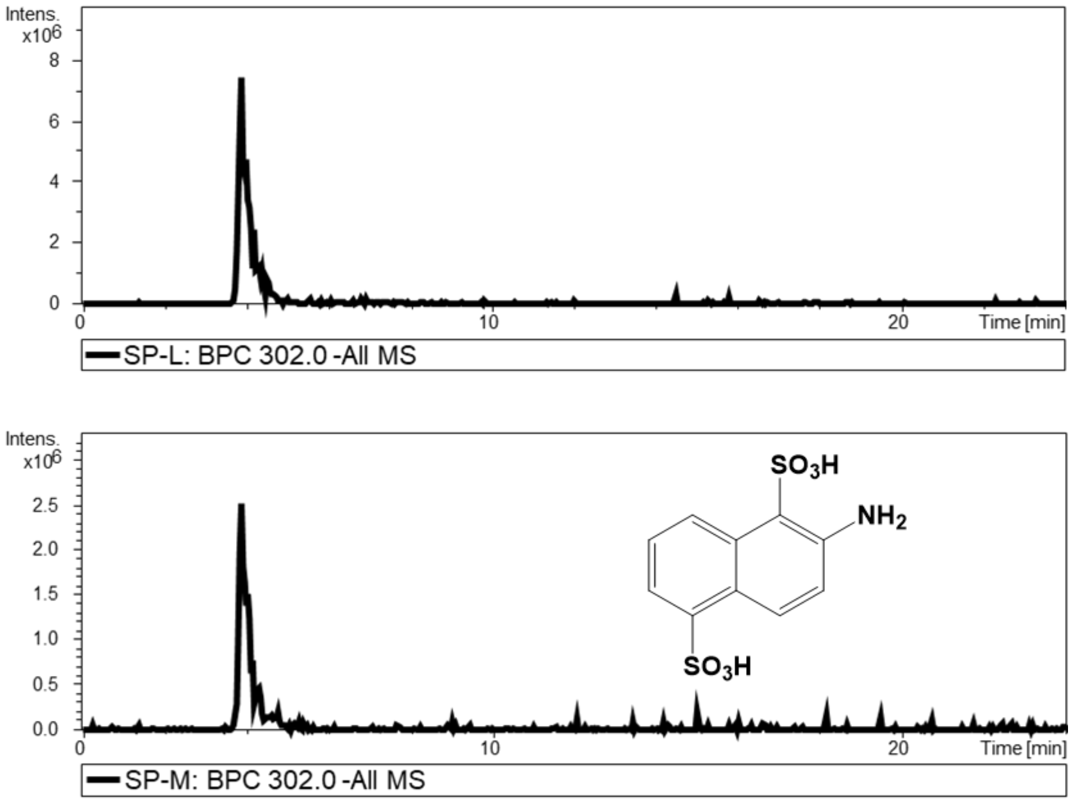

L1

M1

Figure S2. LC-ESI-MS/MS chromatogram of suspicious samples (**A1-M1**) after reductive cleavage ($SnCl_2$/HCl).

Table S2. LOD/LOQ of cleavage products.

Cleavage product	LOD	LOQ
2-amino-1,5-naphthalene-disulfonic acid	9 µg/kg	26 µg/kg
2-amino-1-naphthalene-sulfonic acid	14 µg/kg	46 µg/kg
2-amino-benzene-sulfonic acid	12 µg/kg	35 µg/kg
2-((4-amino-phenyl) sulfonyl)-ethyl-hydrogen sulfate	5 µg/kg	18 µg/kg
